# LncRNA ABHD11‐AS1 promotes the development of endometrial carcinoma by targeting cyclin D1

**DOI:** 10.1111/jcmm.13675

**Published:** 2018-05-25

**Authors:** Yao Liu, Li‐Li Wang, Shuo Chen, Zhi‐Hong Zong, Xue Guan, Yang Zhao

**Affiliations:** ^1^ Department of Obstetrics and Gynecology The Third Affiliated Hospital of Guangzhou Medical University, Key laboratory for Major Obstetric Diseases of Guangdong Province, and Key Laboratory of Reproduction and Genetics of Guangdong Higher Education Institute in Guangdong Province Guangzhou 510150 China; ^2^ Department of Gynecology The First Affiliated Hospital of China Medical University Shenyang 110001 China; ^3^ Department of Biochemistry and Molecular Biology College of Basic Medicine, China Medical University Shenyang 100013 China

**Keywords:** cyclin D1, endometrial carcinoma, LncRNA ABHD11‐AS1, tumorigenesis and progression

## Abstract

To investigate the expression, role and mechanism of action of long non‐coding RNA (lncRNA) ABHD11‐AS1 in endometrial carcinoma. The expression of lncRNA ABHD11‐AS1 was quantified by qRT‐PCR in human endometrial carcinoma (n = 89) and normal endometrial tissues (n = 27). LncRNA ABHD11‐AS1 was stably overexpressed or knocked‐down in endometrial carcinoma cell lines to examine the cellular phenotype and expression of related molecules. Compared to normal endometrial tissue, lncRNA ABHD11‐AS1 was significantly overexpressed in endometrial carcinoma. Overexpression of lncRNA ABHD11‐AS1 promoted the proliferation, G1‐S progression, invasion and migration of endometrial cancer cells; inhibited apoptosis; up‐regulated cyclin D1, CDK1, CDK2, CDK4, Bcl‐xl and VEGFA; and down‐regulated p16, while ABHD11‐AS1 down‐regulation has the opposite effect. RNA pull down demonstrated that lncRNA ABHD11‐AS1 binds directly to cyclin D1. Knockdown of cyclin D1 can reverse the effect of ABHD11‐AS1. Overexpression of lncRNA ABHD11‐AS1 increased the tumorigenicity and up‐regulated cyclin D1 in an in vivo model of endometrial cancer in nude mice. LncRNA ABHD11‐AS1 functions as an oncogene to promote cell proliferation and invasion in endometrial carcinoma by positively targeting cyclin D1.

## 1 INTRODUCTION

The incidence of endometrial cancer is increasing year by year. Endometrial cancer is the second leading cause of deaths due to gynaecological cancer worldwide.^1^ Early‐stage endometrial cancer has a high survival rate, while approximately 30% of patients are diagnosed with advanced stage disease.^2^

Long non‐coding RNAs (lncRNAs) are a class of RNA molecules over 200 nucleotides long without protein‐coding ability that are expressed in a wide range organisms and tissues.^3^, ^4^ LncRNAs represent a new frontier in molecular biology and play significant roles in epigenetic, transcriptional and post–transcriptional regulation of gene expression, are involved in normal cell physiology and participate in the development of various diseases, including cancer.^5^ The lncRNA ABHD11‐AS1 has been found to be overexpressed in gastric cancer,^6^ ovarian cancer^7^ and bladder cancer tissues.^8^ However, the role of ABHD11‐AS1 in endometrial cancer has not been reported. Therefore, we investigated the expression, function and mechanism of action of lncRNA ABHD11‐AS1 in endometrial carcinoma.

## 2 MATERIALS AND METHODS

### 2.1 Tissue specimens

Twenty‐seven normal endometrial specimens that were collected from the normal endometrial tissue of patients with uterine fibroids undergoing hysterectomy, and 89 endometrial carcinomas were collected from patients undergoing surgical resection at the First Affiliated Hospital of China Medical University (Shenyang, Liaoning, China). No patients received pre‐operative chemotherapy or radiotherapy. All specimens were confirmed by two pathologists. This research programme (No. 2016‐32‐2) was approved by the Chinese Medical University Ethics Committee. All tissue samples were processed in accordance with ethical and legal standards.

### 2.2 Cell culture and transfection

Human HEC‐1B and Ishikawa endometrial cancer cell lines were cultured in Dulbecco's modified Eagle's medium supplemented with penicillin/streptomycin (100 U/mL) and 10% foetal bovine serum (FBS; HyClone, Logan, UT, USA) or RPMI‐1640 (HyClone). Ishikawa cells were purchased from Nanjing KeyGen Biotech (Nanjing, China), and HEC‐1B cells were purchased from the China Center for Type Culture Collection (CCTCC, Wuhan, China). Cells were incubated in a 5% CO_2_ incubator at 37°C and passaged routinely. Plasmids and siRNAs (sense: 5′‐GCUACGAGAUCAUGAGCCAdTdT‐3′ and anti‐sense: 5′‐UGGCUCAUGAUCUCGUAGCdTdT‐3′) were transfected into cells using Lipofectamine 2000 (Invitrogen, Carlsbad, USA) following the manufacturer's instructions. The target sequences of cyclin D1 siRNA were 5′‐GUUCAGAAACUAAUCCAGAdTdT‐3′(sense) and 5′‐UCUGGAUUAGUUUCUGAACdTdT‐3′ (anti‐sense). The ABHD11‐AS1 sequence could be found in the Table S3.

### 2.3 Cell proliferation assays

Cells were trypsinized, seeded into 96‐well plates at a density of 3000 cells/well, allowed to adhere, transfected and cultured for 0, 24, 48 or 72 hours. After adding 20 μL of MTT (5 mg/mL), the cells were incubated at 37°C for 2‐4 hours, the medium was discarded, and 150 μL of dimethyl sulphoxide (DMSO) was added under dark conditions. Finally, the OD values were measured at 490 nm using a spectrophotometer (BioTek Instruments, Winooski, VT, USA). Each experiment was performed using triplicate wells (at least).

### 2.4 Cell cycle assays

Control and transfected cells were cultured in six‐well plates for 48 hours, trypsinized, centrifuged at 231 g for 5 minutes, washed twice with PBS; 70% of ice‐cold ethanol was added, incubated at −20°C for 2 hours or overnight, washed twice with PBS; cell cycle detection kit (BD, New Jersey, USA) was added, incubated for 30 minutes at 4°C; and cell cycle analysis was performed by flow cytometry.

### 2.5 Apoptosis assays

Quantification of apoptosis was performed using flow cytometry after staining with annexin V‐labelled with 7AAD and PE (BD Biosciences) according to the manufacturer's instructions. Cells were harvested 48 hours after transfection, washed twice with cold PBS, resuspended, and then a mixture of 100 μL of 1 × Binding Buffer and 5 μL of equal amounts of Annexin V‐PE and 7AAD was added. After incubation in the dark for 15 minutes, 400 μL of 1× Binding Buffer was added, and the cells were analysed by flow cytometry within 1 hour.

### 2.6 Wound‐healing assay

Cells were seeded in six‐well plates at a density of 10^6^ per well, allowed to adhere, and scratches were created in the monolayers using 200‐μL pipette tips, then the cells were washed three times with PBS and cultured in FBS‐free medium with mitomycin C (20 ug/mL), and after the transfection in photomicrographs was captured in the same positions at 0, 24 and 48 hours. The rate of cell migration was analysed using Image J software (National Institutes of Health, Bethesda, MD, USA) as scratch healing rate = (original wound diameter ‐ wound diameter at different time‐points)/original wound diameter × 100%.

### 2.7 Cell invasion assays

Matrigel transwell cell culture chambers (BD Biosciences, San Jose, CA, USA) were used for cell invasion assays. Briefly, 30 μL of the matrix was diluted 1:15, placed into the upper chamber of the Transwell chamber and incubated at 37°C for 4 hours. Cells (5 × 10^4^) were suspended in 200 μL of FBS‐free culture medium and added to the upper chamber; 600 μL of complete culture medium containing 10% FBS was added to the lower chamber. After incubation for 48 hours at 37°C, the cells were washed three times with PBS, fixed with 4% paraformaldehyde, and the matrix and residual cells in the upper chamber were removed with a cotton swab. The cells in the lower chamber were stained with crystal violet, washed three times with PBS, sealed with resin adhesive film, imaged using an Olympus fluorescence microscope (Tokyo, Japan), and the numbers of cells in five independent fields of view in each well were counted.

### 2.8 Real‐time PCR

Tissue specimens or cells were placed into microcentrifuge tubes with 1 mL of TRIzol (Takara, Shiga, Japan), the tissues were cut finely, and then the samples were shaken for 30 seconds. Chloroform was added, mixed thoroughly, incubated at room temperature for 10‐15 minutes and centrifuged at 14803 g for 20 minutes. The upper layer (500 μL) was transferred to a new microcentrifuge tube; an equal volume of isopropyl alcohol was added, incubated at −20°C for 30 minutes and centrifuged at 14803 g for 20 minutes; the supernatant was discarded; 1 mL of 75% ethanol was added and centrifuged at 5782 g for 20 minutes; the supernatant was discarded; and the pellet was air‐dried and dissolved in 10 μL of DEPC water. One microlitre of RNA was added to 79 μL of DEPC water, and the OD260/OD280 ratio was assessed to determine RNA concentration and quality.

Total RNA was reverse transcribed into cDNA using avian myeloblastosis virus transcriptase and random primers (Takara, Shiga, Japan) according to the manufacturer's instructions. The target gene was amplified by real‐time quantitative PCR with SYBR Premix Ex Taq™ II kit (Takara). The relative expression of the target genes was determined by comparing the threshold cycle (*C*t) values of the target genes to that of 18S rRNA (18S) using the 2^−ΔΔ*C*t^ method (GenePharma).

### 2.9 Western blotting

Tissue or cell protein samples were extracted, quantified and diluted with 5× loading buffer to the same concentrations, denatured at 95°C, then separated by electrophoresis on 10% or 12% sodium dodecyl sulphate (SDS) polyacrylamide gels. The proteins were transferred to Hybond membrane (Amersham, Munich, Germany); the membranes were blocked with 5% skimmed milk at room temperature for 1‐2 hours, then incubated with primary antibodies against cyclin D1, CDK1, CDK2, CDK4, Bcl‐xl, VEGFA, and p16 antibodies (1:1000; Proteintech, Proteintech Group, USA) overnight at 4°C. The membranes were washed three times with Tris‐buffered saline (TBST), incubated with the corresponding secondary antibodies (1:5000) for 2 hours and washed three times with TBST, and the protein bands were visualized using enhanced chemiluminescence reagent (Santa Cruz Biotechnology, Santa Cruz, CA, USA).

### 2.10 RNA pull‐down assays

Streptavidin beads (BEAVER, Suzhou, China) were used to capture biotin‐labelled ABHD11‐AS1; IgG was used as a control. The biotinylated nucleic acid compound was incubated with protein lysates prepared from Ishikawa cells transfected with ABHD11‐AS1 for 40 minutes in a 42°C water bath to specifically capture ABHD11‐AS1 and the corresponding bound proteins. After elution of the beads, the protein samples were detected by Western blotting; the transfected protein in the cell samples was used a positive control; and IgG was used as a negative control.

### 2.11 In vivo tumorigenesis model

Ishikawa cells (1 × 10^7^) were resuspended in 150 μL of culture medium without FBS and subcutaneously injected into the right flanks of 4‐week‐old nude mice. The experimental group (5 mice per group) was injected with Ishikawa cells overexpressing ABHD11‐AS1, while the control group was injected with mock‐transfected cells. The mice were housed in a specific sterile environment suitable and regularly observed. Tumour volume was measured every 0.5 weeks, and mice were killed after 8 weeks. The animal experiments were approved by the China Medical University Animal Care and Use Committee and performed in strict accordance with national animal experimental criteria and standards.

### 2.12 Statistical analysis

Data were analysed using SPSS 17.0 statistical software (SPSS Inc., Chicago, IL, USA). Correlations were analysed using Spearman's correlation test. Two‐tailed *t* tests were used to compare mean values. At least three replicates were performed for each group; at least three independent experiments were performed for each assay. All data are expressed as mean ± standard deviation; *P *<* *.05 was considered significant.

## 3 RESULTS

### 3.1 LncRNA ABHD11‐AS1 is overexpressed in endometrial carcinoma

**Figure 1:**
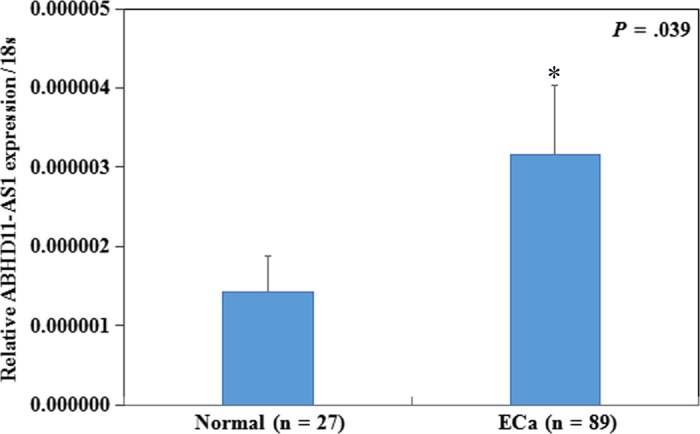
LncRNA ABHD11‐AS1 is overexpressed in endometrial carcinoma. ABHD11‐AS1 expression was significantly higher in endometrial cancer than in normal endometrial tissues (A). *P <* *.05

Real‐time PCR revealed the expression of ABHD11‐AS1 was significantly higher in endometrial carcinoma than in normal endometrial tissues (Figure 1A, *P* < .05).

### 3.2 LncRNA ABHD11‐AS1 promotes proliferation, cycle, inhibits apoptosis and enhances the invasive and metastatic potential of endometrial cancer cells

**Figure 2:**
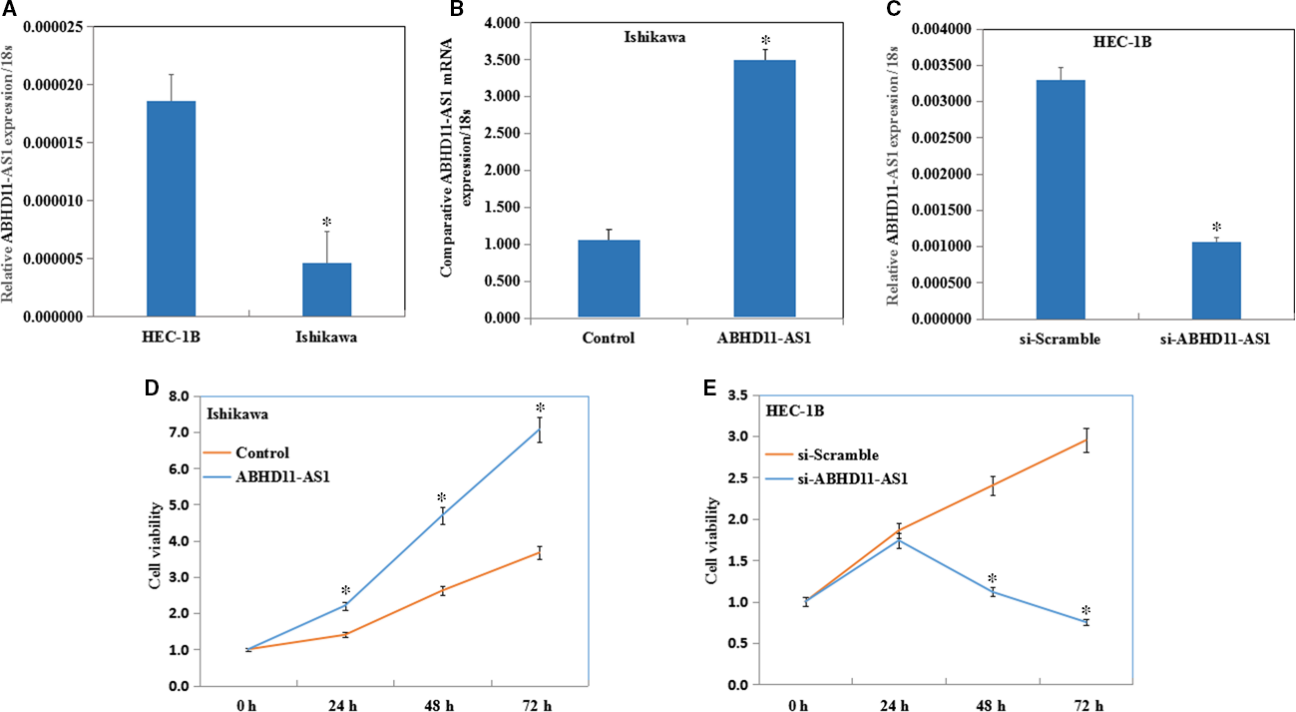
LncRNA ABHD11‐AS1 promotes proliferation of endometrial cancer cells. The expression of ABHD11‐AS1 was lower in Ishikawa cells than HEC‐1B cells (A). After ABHD11‐AS1 transfection, the expression of ABHD11‐AS1 was confirmed by real‐time PCR (B and C). Overexpression of ABHD11‐AS1 significantly increased the proliferation of Ishikawa cells (D), whereas knockdown of ABHD11‐AS1 decreased the proliferation of HEC‐1B cells (E). *P <* *.05

The expression of ABHD11‐AS1 was lower in Ishikawa cells than HEC‐1B cells (Figure 2A, *P *< .05); thereby, Ishikawa cell line was used for ABHD11‐AS1 plasmid transfection, and HEC‐1B cell line was used for si‐ABHD11‐AS1, respectively. The expression of ABHD11‐AS1 was confirmed by real‐time PCR (Figure 2B,C, *P* < .05).

The MTT assay revealed that overexpression of ABHD11‐AS1 significantly increased the proliferation of Ishikawa cells (Figure 2D, *P* < .05), whereas knockdown of ABHD11‐AS1 using the siRNA significantly decreased the proliferation of HEC‐1B cells (Figure 2E, *P* < .05).

**Figure 3:**
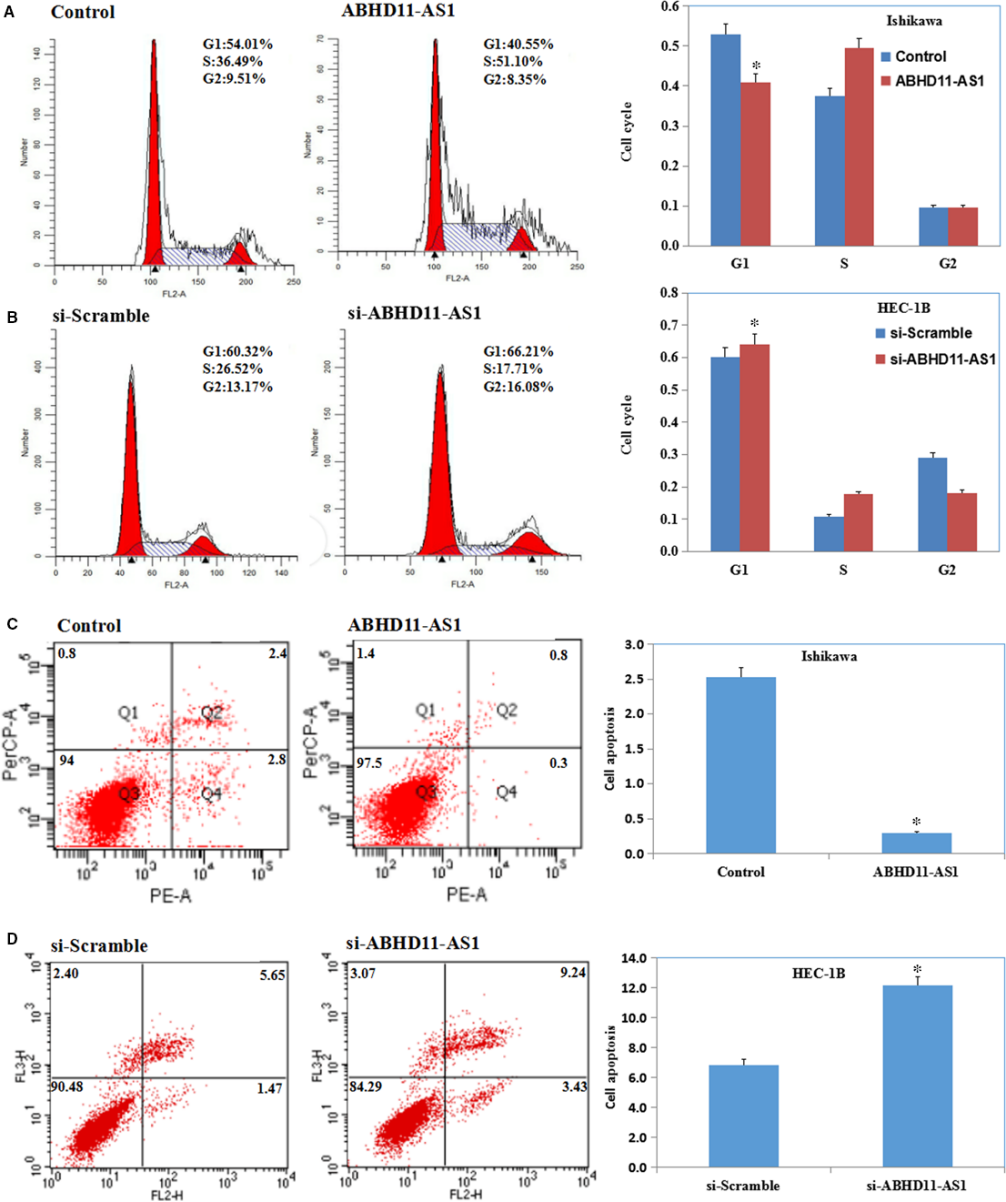
LncRNA ABHD11‐AS1 promotes cycle and inhibits apoptosis of endometrial cancer cells. Cell cycle assays showed that ABHD11‐AS1 increased G1‐S progression in Ishikawa cells (A) and si‐ABHD11‐AS1 induced G1 phase arrest in HEC‐1B cells (B). Apoptosis assays showed that overexpression of ABHD11‐AS1 significantly decreased apoptosis (C), while knockdown of ABHD11‐AS1 significantly increased apoptosis (D). *P <* *.05

Cell cycle assays showed that ABHD11‐AS1 promoted G1‐S progression in Ishikawa cells (Figure 3A, *P* < .05) and si‐ABHD11‐AS1 significantly induced G1 phase arrest in HEC‐1B cells (Figure 3B, *P* < .05).

Apoptosis assays showed that overexpression of ABHD11‐AS1 significantly decreased apoptosis in Ishikawa cells (Figure 3C, *P* < .05), while knockdown of ABHD11‐AS1 significantly increased apoptosis in HEC‐1B cells (Figure 3D, *P* < .05).

**Figure 4:**
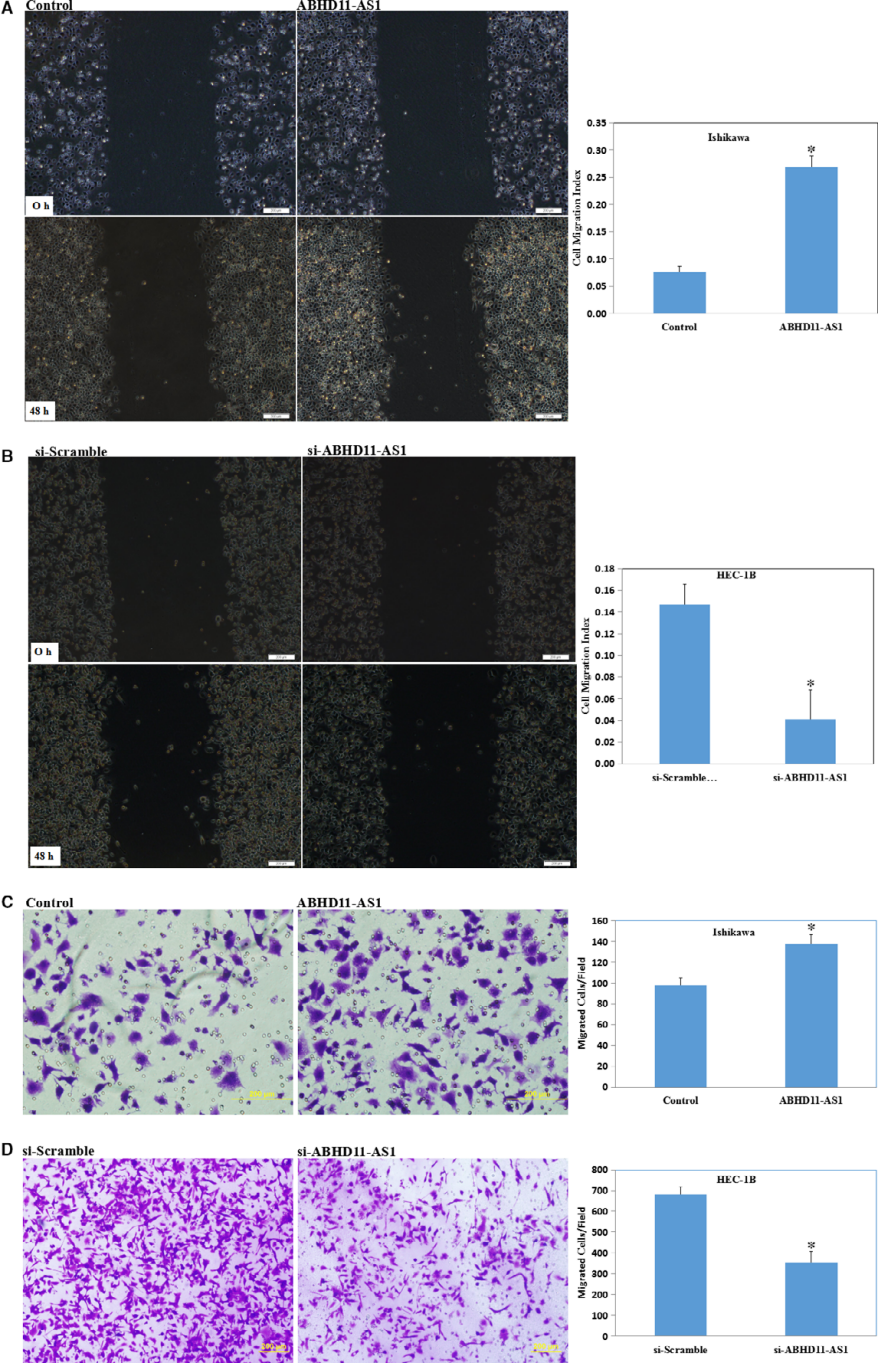
LncRNA ABHD11‐AS1 enhances the invasive and metastatic potential of endometrial cancer cells. Overexpression of ABHD11‐AS1 increased the migratory and invasive ability of Ishikawa cells (A and C), whereas silencing of ABHD11‐AS1 reduced the migratory and invasive ability of HEC‐1B cells compared with the respective vector control cells (B and D). *P <* *.05

The wound‐healing assay and the Transwell assay revealed that ABHD11‐AS1 transfection increased the migratory ability and invasive ability (Figure 4A,C, *P* < .05), whereas si‐ABHD11‐AS1 transfection reduced the migratory ability and invasive ability (Figure 4B,D, *P* < .05).

### 3.3 LncRNA ABHD11‐AS1 promotes endometrial tumorigenicity in vivo

**Figure 5:**
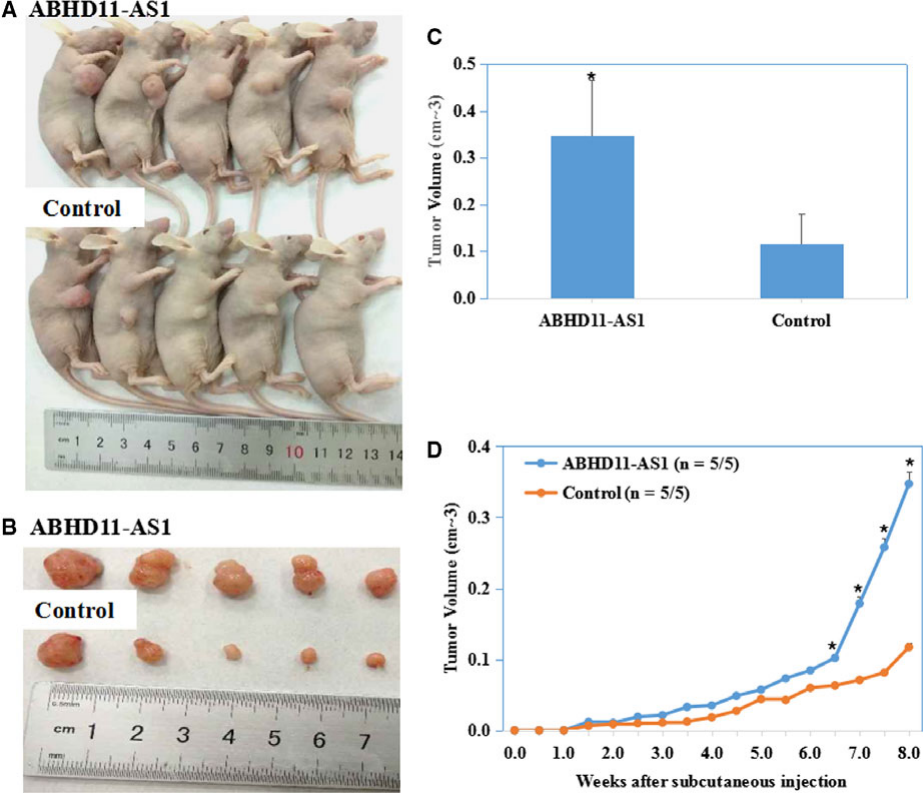
LncRNA ABHD11‐AS1 promotes endometrial tumorigenicity in vivo. The tumour growth rate and size of the tumours in mice injected with cells overexpressing ABHD11‐AS1 group were significantly higher than those of the mice injected with the vector control cells (A‐D). *P <* *.05

Tumour xenograft volume in nude mice treated with ABHD11‐AS1 was greater than that in mock nude mice (Figure 5A,B,C, *P* < .05). The growth rate was also faster than that in the mock group (Figure 5D, *P* < .05).

### 3.4 LncRNA ABHD11‐AS1 up‐regulates cyclin D1, CDK1, CDK2, CDK4, Bcl‐xl and VEGFA and down‐regulates p16

**Figure 6:**
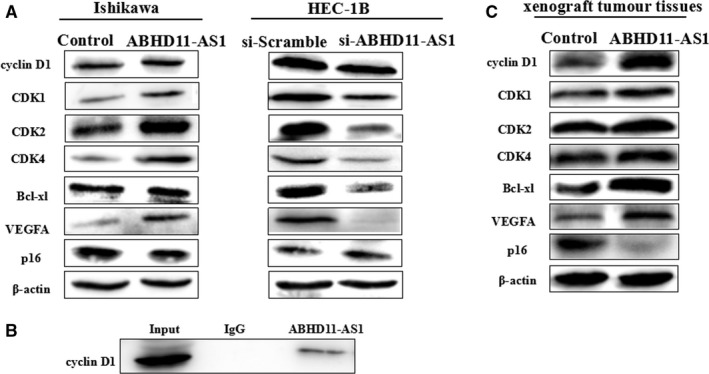
ABHD11‐AS1 is co‐immunoprecipitated with cyclin D1. Overexpression of ABHD11‐AS1 increased the expression of cyclin D1, CDK1, CDK2, CDK4, Bcl‐xl and VEGFA and decreased the expression of p16; conversely, the opposite results were observed in HEC‐1B cells transfected with si‐ABHD11‐AS1 (A). RNA pull‐down assays showed that lncRNA ABHD11‐AS1 is co‐immunoprecipitated with cyclin D1 (B). Western blotting of the xenograft tumour tissues from the nude mouse model showed that overexpression of ABHD11‐AS1 increased the expression of cyclin D1, CDK1, CDK2, CDK4, Bcl‐xl and VEGFA and decreased the expression of p16 (C)

The protein expression levels of ABHD11‐AS1‐overexpressing Ishikawa cells were assessed by Western blotting. Overexpression of ABHD11‐AS1 increased the expression of cyclin D1, CDK1, CDK2, CDK4, Bcl‐xl and VEGFA and decreased the expression of p16 (Figure 6A). Conversely, the opposite results were observed in HEC‐1B cells transfected with si‐ABHD11‐AS1 (Figure 6A).

### 3.5 LncRNA ABHD11‐AS1 is co‐immunoprecipitated with cyclin D1

RNA pull‐down assays were performed to detect the protein that interacted with ABHD11‐AS1. Protein from RNA pull‐down assays with biotin‐labelled ABHD11‐AS1 against ABHD11‐AS1 was used for Western blot, which demonstrated an enrichment of cyclin D1 compared with IgG (Figure 6B). Western blotting of the xenograft tumour tissues from the nude mouse model showed that overexpression of ABHD11‐AS1 increased the expression of cyclin D1, CDK1, CDK2, CDK4, Bcl‐xl and VEGFA and decreased the expression of p16 (Figure 6C).

### 3.6 LncRNA ABHD11‐AS1 promotes the development of endometrial cancer by targeting cyclin D1

**Figure 7:**
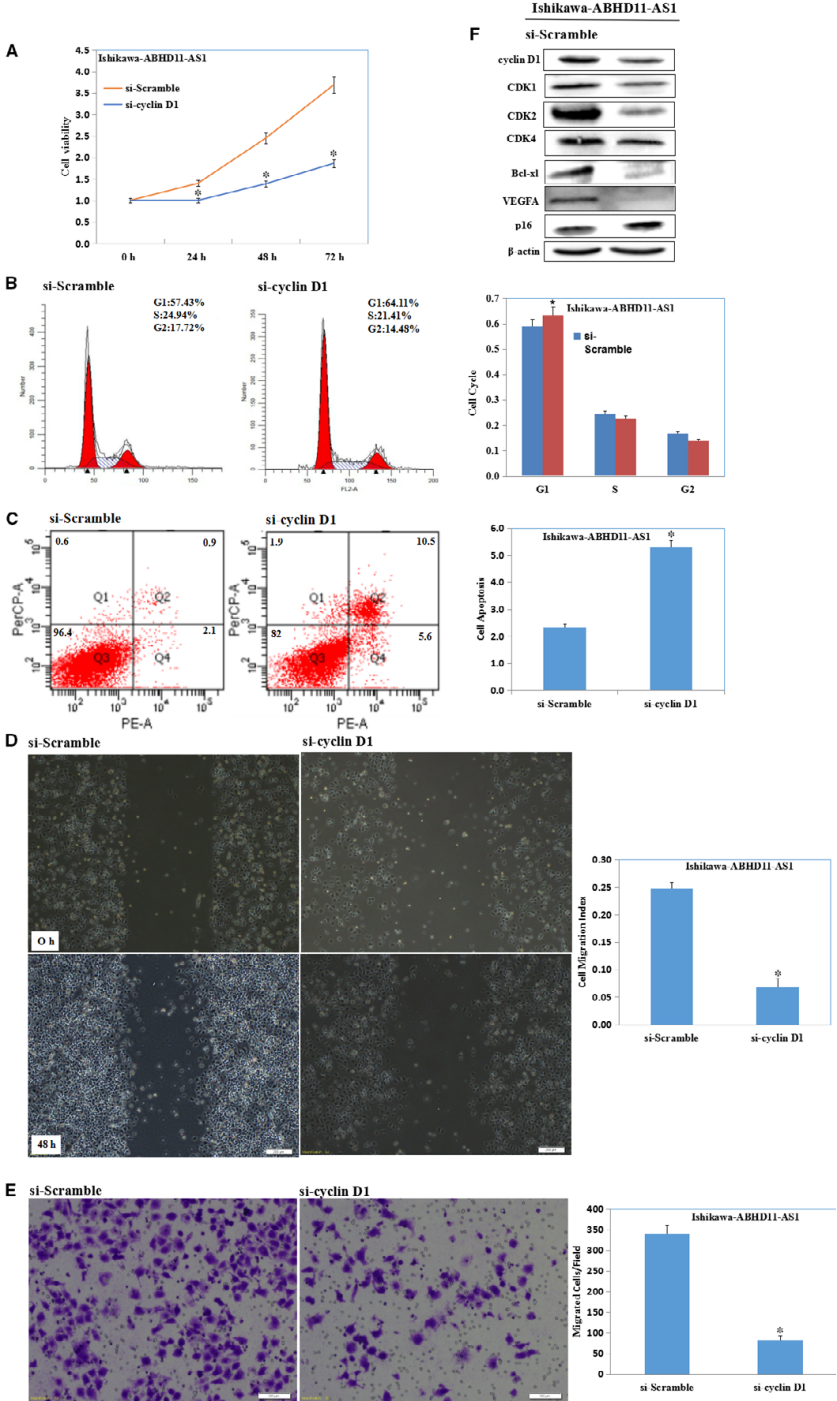
LncRNA ABHD11‐AS1 promotes the development of endometrial cancer by targeting cyclin D1. Silencing of cyclin D1 in ABHD11‐AS1‐overexpressing Ishikawa cells significantly inhibited proliferation (A), induced G1 phase arrest (B) and apoptosis (C), inhibited migration (D) and invasion (E). Western blotting showed that silencing of cyclin D1 reversed the ability of ABHD11‐AS1 to up‐regulate cyclin D1, CDK1, CDK2, CDK4, Bcl‐xl and VEGFA and down‐regulate p16 expression (F)

A siRNA was used to silence cyclin D1 in ABHD11‐AS1‐overexpressing Ishikawa cells; Silencing of cyclin D1 in ABHD11‐AS1‐overexpressing Ishikawa cells significantly inhibited proliferation (Figure 7A, *P* < .05), induced G1 phase arrest (Figure 7B, *P* < .05) and apoptosis (Figure 7C, *P* < .05), inhibited migration (Figure 7D, *P* < .05) and invasion (Figure 7E, *P* < .05). Furthermore, Western blotting showed that silencing of cyclin D1 reversed the ability of ABHD11‐AS1 to up‐regulate cyclin D1, CDK1, CDK2, CDK4, Bcl‐xl and VEGFA and down‐regulate p16 expression (Figure 7F).

## 4 DISCUSSION

LncRNAs have emerged as a hot spot of research and been closely associated with human cancer in recent years. Numerous studies have shown lncRNAs are differentially expressed in normal and tumour tissues,^9^ including endometrial cancer.^10^, ^11^, ^12^, ^13^ Numerous lncRNAs that are altered in cancer have been linked to the proliferation and invasion of tumour cells and may represent candidate biomarker molecules for the diagnosis and treatment of cancer. LncRNA ABHD11‐AS1 is overexpressed and associated with tumour size and stage in gastric cancer and may have potential as a screening biomarker for gastric cancer.^6^ ABHD11‐AS1 is also overexpressed in ovarian cancer and bladder cancer,^7^, ^8^ and can promote proliferation and invasion and inhibit apoptosis in ovarian cancer and bladder cancer cells, thus may represent a potential target for bladder cancer. Therefore, we assessed the expression and role of ABHD11‐AS1 in endometrial cancer.

We found ABHD11‐AS1 was significantly up‐regulated in 89 endometrial cancer tissues compared with normal endometrial tissues. Therefore, ABHD11‐AS1 may function as an oncogene during the development of endometrial cancer. In agreement with this hypothesis, overexpression of ABHD11‐AS1 promoted proliferation, G1‐S progression, migration and invasion and inhibited apoptosis in Ishikawa cells. Conversely, silencing ABHD11‐AS1 in HEC‐1B cells had the opposite effects. Moreover, a tumorigenesis assay demonstrated overexpression of ABHD11‐AS1 promoted endometrial cancer cell tumorigenicity and tumour growth in vivo in nude mice. These observations are consistent with the reported roles of ABHD11‐AS1 in gastric cancer, ovarian cancer and bladder.^6^, ^7^, ^8^

LncRNAs participate in various physiological and pathological processes, including epigenetic regulation and the development of cancer. In addition, lncRNAs have the unique ability to interact directly with both nucleic acids and proteins and have discontinuous effects on many biological processes.^4^ Several studies have shown lncRNAs play important roles in the regulation of protein expression.^10^, ^11^, ^12^, ^14^ In this study, Western blotting revealed overexpressing ABHD11‐AS1 increased the protein levels of cyclin D1, CDK1, CDK2, CDK4, Bcl‐xl and VEGFA and decreased the levels of p16, both in vivo and in vitro. Alterations to cyclin D1, CDK1, CDK2, CDK4 and p16, which regulate the cell cycle, led us to speculate whether ABHD11‐AS1 affects tumour progression by regulating the protein expression of factors linked to the cell cycle. Interestingly, we detected the downstream binding protein of ABHD11‐AS1 by RNA pull‐down assays. Fortunately, we found that ABHD11‐AS1 binds directly to cyclin D1 and targets cyclinD1 to play a biological role. Western blotting confirmed overexpression of ABHD11‐AS1 increased cyclin D1 expression in the xenograft tumours.

Cyclins play key roles in cell proliferation and growth.^15^ Cyclin D1, one of the three mammalian cyclin D molecules, is the most well‐studied cyclin. Cyclin D1 is more frequently differentially expressed in human cancers than cyclin D2 or D3, is an important mediator of cancer initiation, development and metastasis and is associated with poor prognosis.^15^, ^16^, ^17^, ^18^, ^19^ Cyclin D1 is widely involved in cancer: cyclin D1 promotes cell proliferation in colorectal cancer^20^; EMSY and CCND1 work together to contribute to the pathogenesis of lung cancer^21^; extracellular adenosine induces G1 cell cycle arrest via the cyclin D1/CDK4 pathway in ovarian cancer^22^; the EGFR/PI3K/Akt/cyclin D1 signalling pathway may play a key role in cholesteatoma^23^; microRNA‐338‐3p can bind cyclin D1 and mediates hepatocyte proliferation in hepatocellular carcinoma^24^, ^25^; P16/cyclin D1/CDK4 signalling pathway alterations may affect epithelial cell proliferation and apoptosis in gallbladder carcinoma, even during initiation^26^; and β‐catenin is involved in pancreatic cancer carcinogenesis and metastasis by up‐regulating cyclin D1.^27^

Furthermore, silencing cyclin D1 in Ishikawa cells stably transfected with ABHD11‐AS1 reversed the ability of ABHD11‐AS1 to promote cell proliferation, G1‐S progression and invasion, inhibit apoptosis, up‐regulate cyclin D1, CDK1, CDK2, CDK4, Bcl‐xl and VEGFA and down‐regulate p16. These results suggest ABHD11‐AS1 regulates cell proliferation, cycle, apoptosis and invasion in endometrial cancer—at least in part—by targeting cyclin D1 and in turn the expression of CDK1, CDK2, CDK4, Bcl‐xl, VEGFA and p16.

In conclusion, this study reveals ABHD11‐AS1 is up‐regulated and promotes tumorigenesis in endometrial carcinoma by positively targeting cyclin D1. This is the first demonstration of an oncogenic role for ABHD11‐AS1 in endometrial carcinoma; this finding could potentially pave the way for the development of novel strategies for diagnosis and treatment.

## ETHICS APPROVAL AND CONSENT TO PARTICIPATE

The research protocol was approved by the China Medical University Ethics Committee (No: 2016‐32‐2).

## CONSENT FOR PUBLICATION

Not applicable.

## AVAILABILITY OF DATA AND MATERIALS

The data sets used and/or analysed during the current study available from the corresponding author on reasonable request.

## CONFLICT OF INTERESTS

The authors have no conflict of interests to declare.

## AUTHORS’ CONTRIBUTIONS

Yang Zhao conceived the study and analysed interpretation. Yao Liu performed the experiments, analysed data and wrote the manuscript. Li‐Li Wang, Shuo Chen, Zhi‐Hong Zong and Xue Guan performed the experiments and analysed the data. All authors read and approved the final manuscript.
