# Supplementary material for: LncRNA ABHD11‐AS1 promotes the development of endometrial carcinoma by targeting cyclin D1
Source: J Cell Mol Med. 2018 May 25;22(8):3955–64. doi: 10.1111/jcmm.13675 (PMC6050509; doi:10.1111/jcmm.13675)
Supplement: Supplementary file 1 [file JCMM-22-3955-s001.doc]

**Supplementary Table 1:** LncRNA ABHD11-AS1 expression in Normal endometrium and Endometrial cancer tissues

| **Groups** | **N** | **LncRNA ABHD11-AS1**  **expression / 18s** | ***P* value** |
| --- | --- | --- | --- |
|
| Normal endometrium | 27 | 1.428E-06 ± 2.312E-06 |  |
| Endometrial cancer | 89 | 3.161E-06 ±8.234E-06 | ***0.039*** |

Bold and Italics means P < 0.05.

**Supplementary Table 2:** Correlation of LncRNA ABHD11-AS1 expression with different clinicopathological features of ovarian carcinoma

| **Clinicopathological features** | **N** | **LncRNA ABHD11-AS1 expression / 18s** | ***P* value** |
| --- | --- | --- | --- |
|
| **The pathology types** |  |  | ***0.008*** |
| Endometrioid adenocarcinoma | 55 | 1.359E-06±3.755E-06 |  |
| The other pathology types | 34 | 6.075E-06±1.198E-05 |  |
| **Age** |  |  | 0.112 |
| ≤ 55 | 38 | 1.989E-06±6.413E-06 |  |
| > 55 | 51 | 4.034E-06±9.331E-06 |  |
| **FIGO stages** |  |  | 0.177 |
| I-II | 67 | 2.533E-06±6.530E-06 |  |
| III-IV | 22 | 5.07E-06±1.204E-05 |  |
| **Pathology classification** |  |  | 0.391 |
| Well | 46 | 3.394E-06 ±9.155E-06 |  |
| Mod + Poor | 43 | 2.911E-06 ±7.218E-06 |  |
| Bold and Italics means P < 0.05. | | | |

**Supplementary Table 3:** The ABHD11-AS1 sequence was CTCGAGTGAAGACGGAAATGGGCGGGGCTGCGAGCTAGGGCGGGAGAAGGAGCGCGGGGAGGACGTACCTTGTGAGATGCGAGCCGGCCAACAGCTTGCAAGCATGCTCCGCTGGACCCGAGCCTGGAGGCTCCCGCGTGAGGGACTCGGCCCCCACGGCCCTAGCTTCGCGAGGGTGCCTGTCGCACCCAGCAGCAGCAGCGGCGGCCGAGGGGGCGCCGAGCCGAGGCCGCTTCCGCTTTCCTACAGGCTTCTGGACGGGGAGGCAGCCCTCCCGGCCGTCGTCTTTTTGCACGGGCTCTTCGGCAGCAAAACTAACTTCAACTCCATCGCCAAGATCTTGGCCCAGCAGACAGGCCGTGCTGACGGTGGATGCTCGTAACCACGGTGACAGCCCCCACAGCCCAGACATGAGCTACGAGATCATGAGCCAGGACCTGCAGGACCTTCTGCCCCAGCTGGGCCTGGTGCCCTGCGTCGTCGTTGGCCACAGCATGGGAGGAAAGACAGCCATGCTGCTGGCACTACAGAGGGTGAGCCGCCCATGTCTGGGGCCTCCTCCCATTCAGTATATACCCTGAGGGCCCTGCAGGCAACCTGGGACTCACATGATCGTTGGATGACCAAGTTCAGGCTCCAGGAGCCATGCCTGAGACTCCCTATGTCTGCCTAAGACTGGTCCCAGTTCGGTTCTCTCCCACAGCCAGAGCTGGTGGAACGTCTCATTGCTGTAGATATCAGCCCAGTGGAAAGCACAGGTGTCTCCCACTTTGCAACCTATGTGGCAGCCATGAGGGCCATCAACATCGCAGATGAGCTGCCCCGCTCCCGTGCCCGAAAACTGGCGGATGAACAGCTCAGTTCTGTCATCCAGGACATGGCCGTGCGGCAGCACCTGCTCACTAACCTGGTAGAGGTAGACGGGCGCTTCGTGTGGAGGGTGAACTTGGATGCCCTGACCCAGCACCTAGACAAGATCTTGGCTTTCCCACAGAGGCAGGAGTCCTACCTCGGGCCAACACTCTTTCTCCTTGGTGGAAACTCCCAGTTCGTGCATCCCAGCCACCACCCTGAGATTATGCGGCTCTTCCCTCGGGCCCAGATGCAGACGGTGCCGAACGCTGGCCACTGGATCCACGCTGACCGCCCACAGGACTTCATAGCTGCCATCCGAGGCTTCCTGGTCTAAGAGTTGCTGGCAAGAAGATGGCCGGGCGTGGTGGCTCATGCCTGTAATTCCAGCACTTTGGGAGGCTAAGGCGGGAGGATGACTTGAGGCCAGGAGTTGGAGACCAGCCTGGCCAACATGGTGAAACCCTGTCTCTACTAAAAATACAAAAATTAGCCTGGCGTGGTGGTGCACACCTGTAATCCCAGCTACTCTGGAGGCTGAGGCAGGAGAATCACTTGAACCCTGGAGGCAGAGGTTGCAATGAGCCGAGATCACACCACTACACTCCAGCCTAGGCAACAGAGCAAGACTCTGTCTCAAAAAAAACAAAACAAAAAGGAGGCACAAAACCCCAGGCTTCAAGTCTCTGCAGCCTGCTCCACATTTGGGCACAGAAGGACTCAGACAGGCACTGTGTGGGCACGAGGTTTTACAGGGGTGGTCAGACCTCAGGCTTTAATGAATAAAGACACTACTCCCAAAGGTACC
